# Electrospinning of Biomedical Nanofibers/Nanomembranes: Effects of Process Parameters

**DOI:** 10.3390/polym14183719

**Published:** 2022-09-06

**Authors:** Balaji Ayyanar Chinnappan, Marimuthu Krishnaswamy, Huaizhong Xu, Md Enamul Hoque

**Affiliations:** 1 Department of Mechanical Engineering, Coimbatore Institute of Technology Coimbatore, Tamilnadu 641014, India; 2 Department of Biobased Materials Science, Kyoto Institute of Technology (KIT), Matsugasaki Hashikamicho, Sakyo-ku, Kyoto 606-8585, Japan; 3 Department of Biomedical Engineering, Military Institute of Science and Technology (MIST), Dhaka 1216, Bangladesh

**Keywords:** electrospinning, process parameter, polymer, nanofiber, tissue engineering

## Abstract

Nanotechnology has attracted great attention from researchers in modern science because nanomaterials have innovative and superior physical, chemical, and biological properties, and they can be altered and modified accordingly. As particles get smaller, their surface area increases compared to their volume. Electrospinning is one of the advanced techniques to produce ultrathin nanofibers and membranes, and it is one of the best ways to create continuous nanomaterials with variable biological, chemical, and physical properties. The produced fibers can be utilized in various domains such as wound dressing, drug release, enzyme immobilization, etc. This review examines the biomedical nanofibers/membranes produced by electrospinning techniques to investigate the effects of process parameters (e.g., solution characteristics, applied voltage, and ambient conditions) on nanofiber characteristics (physical, chemical, and mechanical properties). The solution parameters like (i) optimum concentration, (ii) higher molecular weight, and (iii) higher conductivity produce uniform nanofibers, smoother nanofibers, and a smaller and more uniform fiber diameter, respectively. In addition, process parameters such as (i) higher voltage and (ii) slower flow rate produce more polymer ejection from the nozzle and enhance the smoother fiber production, respectively. The optimum tip-to-collector distance is considered to be 13–15 cm. The ambient conditions such as (i) higher humidity and (ii) higher temperature produce thicker and thinner nanofibers, respectively. The controlled parameters through optimization process determine the size and quality of the fibers. The effects of each parameter are discussed in this review. The applications of nanofibers are also discussed.

## 1. Introduction: Process Overview

**Figure 1:**
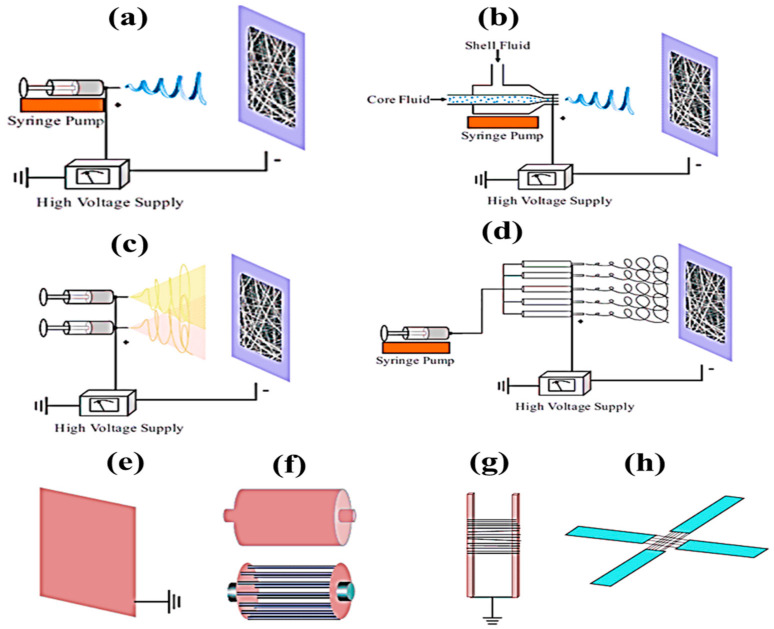
Different types of electrospinning, (**a**) basic, (**b**) co-axial, (**c**) side-by-side, (**d**) multiple-jet, and various types of collector, (**e**) metallic plate, (**d**) drum, (**f**) parallel electrode and (**h**) array of counter electrodes [8].

Recently, nanofibers have been used in a wide range of different applications because of their specific unique features, which include a low density, high surface area to weight ratio, high porosity, small porosity, improved membrane stiffness, and membrane tensile strength. Drawings, phase separation, self-assembly, synthesis of polymeric, and electrospinning are just a few of the ways that may be used to make polymer nanofibers. Only discontinuous nanofibers are formed during the drawing process. To produce fibers of a certain diameter, the template synthesis technique is used. Fewer polymers can be obtained through phase separation. A modest rate of fiber self-assembly is seen in the process. For continuous nanofiber production with variable fiber diameters, electrospinning (ES) is a better option. For aligned fiber, nonwoven fiber, patterned fiber, random three-dimensional structures and micron-sized springs, this procedure may also be utilized to manufacture them. It is possible to create fibers ranging in diameter from 2 nm to several micrometers by combining natural and synthetic polymers, ceramics, solid particle suspensions, liquid crystals, and emulsions. The ES technique used for fabricating nanofibers has attracted increasing attention in the scientific community across the globe. The nanofibers can be used in wider areas including medical, tissue engineering, food packing, water filtration, and so on [1,2,3,4,5,6,7]. ES is a type of nanofiber-spinning technology that relies entirely on the viscoelastic qualities of a high-voltage solution to generate and grow a single charged jet and its deposit on the various collectors depicted in Figure 1, such as (a) basic collector; (b) co-axial collector; (c) side-by-side (two-nozzle electrospinning) collector; (d) multiple-jet collector; (e) metallic-plate collector; (f) drum collectors; (g) parallel-electrode collector; and (h) an array of counter electrodes [8]. The size and shape of the fibers or fiber mats and the convenience to the end user determine the type of electrospinning. The type of electrospinning also decides the quality of the nanofibers.

**Figure 2:**
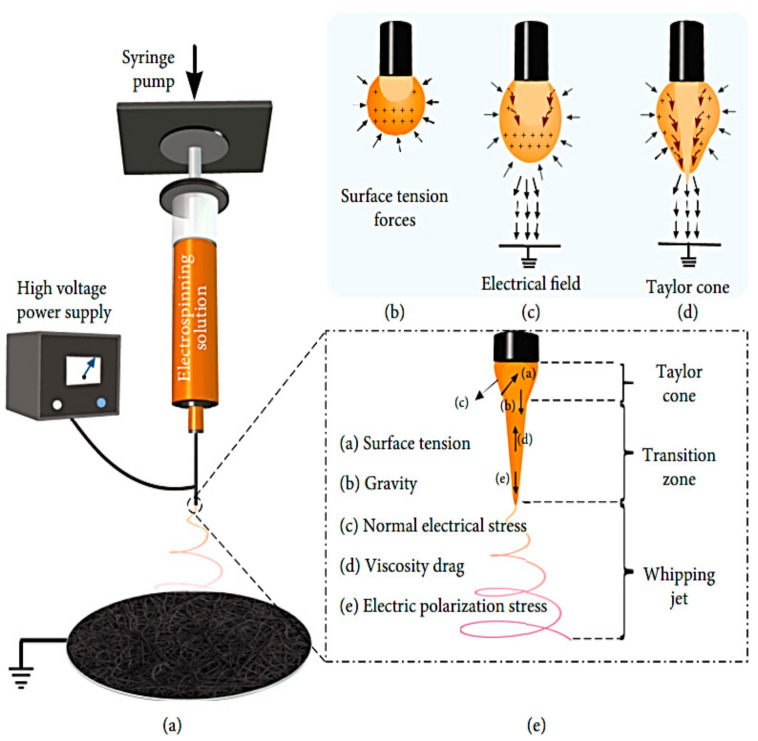
Typical electrospinning process setups [9].

Figure 2 shows a novel nonwoven material with nanofibers ranging in diameter from micrometers to nanometers (a). This process begins with an electrically charged thin nano-sized jet of liquid, with the viscous solution being ejected onto a collector plate from an initially spherical droplet (Taylor cone) in a tiny conical form (Figure 2b–d). Using an ES setup, a nozzle disperses a viscous liquid solvent across a fiber matrix until a fiber solidifies on a grounded plate. Figure 2e shows how the flow rate, number of collectors, and distance between the collector and tip impact the fibers’ shape and diameter (macro to nano) when placed on collectors after the curing process [9]. As the ES temperature increases, the viscosity of the polymeric solution drops, which promotes and enhances droplet formation. Incomplete evaporation of the newly produced polymeric clear liquid solution and fiber solidification might result if the relative humidity of the liquid solution increases. This decreases the amount of evaporation of the fibroin solvent [10]. An increase in the diameter of the nanoparticles leads to a decrease in the diameter of the corresponding electrospun fiber. The shear viscosity increased with a decrease in the diameter of the suspended nanoparticle in spite of using constant-nanoparticle-weight fractions in preparing the suspensions [11].

In this paper, we studied the generation of polymer-free electrospun nanofibrous webs of vitamin-A acetate/CD inclusion complexes which have freestanding, flexible, lightweight, and foldable character, and we aimed to integrate the unique properties of both cyclodextrin inclusion complexes and the electrospun nanofiber to develop a new generation food/dietary supplements with orally fast-dissolving properties and nanofibrous web structure [12]. Flexible and self-standing hydroxypropyl-β-cyclodextrin/difenoconazole inclusion complex (HPβCD/DZ-IC) nanofibers were prepared by polymer-free electrospinning, which exhibited potential to be a new fast-dissolving pesticide formulation [13]. The review article aims to discuss the different methods of electrospinning and critically analyzes the effect of significant parameters such as (i) process, (ii) solution, (iii) and ambient parameters of the electrospinning process and its applications.

### Methods of Electrospinning

**Figure 3:**
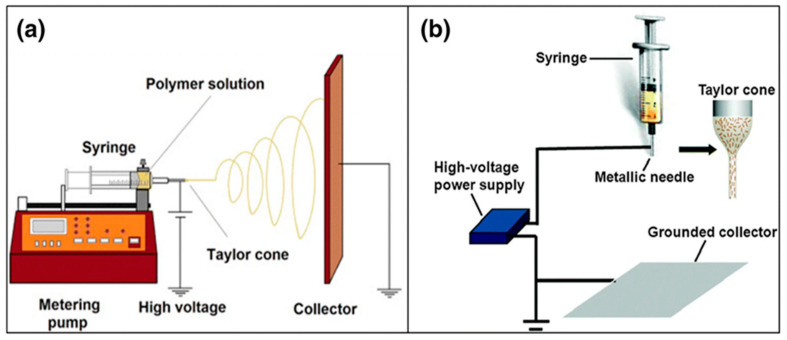
(**a**) HES setup and (**b**) VES setup [14].

The pump is situated parallel to the ground when using the horizontal electrospinning (HES) arrangement shown in Figure 3a. The various kinds of collectors are put across the syringe’s needle. Vertical electrospinning (VES) is a technique in which an electrostatic field is generated perpendicular to the ground floor by a polymeric solution’s vector shown in Figure 3b. This is called horizontal electrospinning since it produces an electric field parallel to the floor. The collection is positioned on the insulated bottom level in the vertical ES arrangement, and the clear syringe pump is placed above it. The HES and VES collectors both make use of a flat iron plate with a predetermined surface area. The transparent syringe pump is held across the collecting plate’s center, beginning at its center. When the electrical field vector of the polymeric solution was exactly parallel to the floor in the HES condition, it was discovered that polymeric droplets tended to fly in a projectile direction and motion. Some fibers are gathered from the collector iron plate even when the Taylor cone is oriented upwards, even though the collector iron plate is moving in a projectile motion. The roughness of the needle in the ES setup and the droplet form may be affected by minor imperfections on the syringe pump needle tip [14].

Investigated [15] the ES process parameters (electric field, flow rate, molecular weight, and concentration) for several polymeric solutions (polyurethane, polyacrylic acid, polycaprolactone, polyvinyl alcohol, and polyethylene oxide) and discovered the critical role and effect of the various operational process parameters such as surface, volume and electrical current. Macro/nanofibers are created using electrostatic forces from novel polymer solutions that have never been used before. The straightforward and affordable experimental setup is an appealing feature of the HES and VES process. The three most significant parts are the spinneret (often made of metal), the source of high voltage, and the collector (grounded or negatively biased). It is usual practice to utilize a cylindrical syringe pump to expel a freshly manufactured solution or a mixture of polymers from the spinneret. As part of ES, an electric, freshly manufactured polymeric fluid jet was used to generate and extend new polymer fibers. When the electrostatic force generated by the repulsion of like charges in the polymeric fluid overcomes the surface tension, the new polymeric fluid droplet emerging from the spinneret deforms into a unique conical shape formation known as a Taylor cone, named after Geoffrey Ingram Taylor for his pioneering research on electrified fluids. 

A metallic nozzle or needle injects a new polymeric liquid solution at a consistent feed rate. High voltages of 10 to 30 KV are used (positive or negative). When a sufficiently high voltage is supplied to the polymeric liquid, a charge accumulates on the hemispherical surface of the polymer droplet, forming a Taylor cone. A liquid jet from the charged polymeric is ejected from the freshly created new Taylor cone and attracted to the collector, fixed away from the metallic needle as the voltage increases. The solvents in the newly created clear polymeric solution evaporate during this process, leaving the collector with dry polymer macro- or nanofibers. A series of poly (vinyl alcohol)/chitosan (PVA/CS) electrospun nanofibers with different weight ratios of PVA and CS were fabricated by the electrospinning method.

As a result of PVA and CS composition measurements, the electrospun nanofibers’ morphologies were mainly affected by the weight ratio of the polymer solution. When increasing the chitosan content in the blend solution, the electrospun nanofibers could hardly form. This result indicates that the electrospun nanofiber formation is enhanced by chitosan content [16]. Earlier work has indicated that continuous fibers of approximately uniform diameter, of both PVA and PVA/ZnO hybrids, can be readily fabricated using electrospinning [17]. In addition to their nano-scale dimensions, electrospun nanofibers are of special interest in biomedical applications, as they imitate the native extracellular matrix of physiologic tissues [18].

## 2. Effects of Process Parameters

The solvents used for various polymers with characterizations of prepared electrospun new polymers are listed in Table 1.

The primary classification of electrospinning parameters was divided into three categories and is illustrated in Table 2.

The solution parameters (i) of optimum concentration, (ii) higher molecular weight, and (iii) higher conductivity produce uniform nanofibers, smoother nanofibers, and a smaller and more uniform fiber diameter, respectively. Additionally, process parameters such as (i) higher voltage and (ii) slow flow rate produce more polymer ejection from the nozzle and enhance the smoother fiber production, respectively. The optimum tip-to-collector distance is 13–15 cm. The ambient parameters such as (i) higher humidity and (ii) increasing temperature produce thicker fibers and generate a thinner nanofiber, respectively. The controlled parameter through optimization decides the size and quality of the fibers.

### 2.1. Electrospinning Solution Parameters

#### 2.1.1. Electrospinning Polymeric Solution Concentrations

Fiber production in the ES process is greatly influenced by the freshly synthesized polymeric liquid solution concentration. (i) Micro- or nanopolymeric fibers are produced when the polymeric solution concentration is very low. (ii) On macro- and nanofibers, a combination of beads and fibers will develop because the polymeric solution concentration is somewhat greater. (iii) Smooth macro- and nanofibers may be generated when the optimal and most suitable polymeric solution concentration is used. (iv) Fibers and helix-shaped microribbons will be generated if the polymeric solution concentration is exceptionally high, as illustrated in Table 3 [20,21,22].

The solution concentrations at the four different levels ((i) very low, (ii) a little higher, (iii) optimum, and (iv) very high) had different effects in the fiber that was observed in the microstructure images.

#### 2.1.2. Molecular Weight of the Polymeric Solution

Morphologies of newly synthesized electrospun nanofibers are influenced by the new polymers’ molecular weight and compared in Table 4. When the polymer solution’s molecular weight is reduced, the fixed concentration produces beads rather than smooth fibers. Creating a smooth nanofiber is possible by increasing the polymeric solution’s molecular weight. Microribbon fibers may be produced by increasing the polymeric solution’s molecular weight [23].

The molecular weight of the polymeric solutions plays a vital role and revealed the following different effects from the varying range of concentrations: unstable and with a bead-on-string structure; fibrous structure which is stabilized; flat fibers; microribbon; helical fibers; and zigzag ribbon patterns.

#### 2.1.3. Polymeric Solution: Viscosity

The viscosity of the freshly synthesized polymeric solution determines the structure and morphology of the newly generated fiber. The extremely (i) low viscosity of newly synthesized polymeric solutions could not produce continuous and smooth fibers. In comparison, the extremely (ii) high viscosity of newly synthesized polymeric solutions leads to the jets being rapidly ejected from the solution. When the solution has a viscosity sufficient for electrospinning, it is possible to regulate the viscosity of a freshly synthesized polymeric solution by altering the concentration of the polymeric solution.

The viscosity, polymer content, and molecular weight of a polymeric solution are all related. Surface tension dominates in freshly synthesized polymeric solutions with low viscosity, resulting in beads or beaded fibers. A sufficient viscosity polymeric solution might provide continuous fibers. As the viscosity of a recently synthesized polymeric solution is directly proportional to concentration and surface tension [24,25]. The effects of viscosity with different range decide the fibers’ structures and their length which is to be optimized by trial studies as per the need.

#### 2.1.4. Polymeric Solution Surface Tension

Electrospun product morphologies in ethanol, MC, and DMF were studied in relation to surface tension. In their research, they discovered that various solvents may have an effect on surface tension: i) reducing the surface tension of the solution to smooth out beaded fibers. The mass ratio of liquids and fiber morphologies can affect the surface tension and solution viscosity. The solvent affects the surface tension of the polymeric solutions and deviates the fibers’ shape and size.

#### 2.1.5. Polymeric Solution: Surface Charge Density/Conductivity

The solution conductivity of newly produced polymeric solutions is determined by the polymer, the solvent, and the salt. In general, natural polymers are polyelectrolytic. Due to the increased strain created by the electric field, the ions boost the charge-carrying capacity of the freshly synthesized polymeric jet, resulting in inferior fiber production in comparison to the synthetic equivalent. Additionally, the polymeric solution’s electrical conductivity may be adjusted through incorporating ionic salts such as NaCl and KH_2_PO_4_. The incorporation of ionic salts resulted in the formation of new nanofibers with a tiny diameter.

### 2.2. Process Parameters of Electrospinning

#### 2.2.1. Polymeric Solution: Voltage

In the ES technique, the applied voltage plays a critical role. When the applied voltage surpasses the threshold voltage, Taylor-cone polymeric solution charged jets may form. Increased voltages may enhance the electrostatic repulsive force acting on the charged jet of polymeric solution, resulting in contraction of the diameter of the fiber.

Electrostatic charges are affected by how much voltage is supplied to the polymeric solution. More polymeric solution is sucked from the needle tip as the voltage is raised, speeding up the ES jet. Because of the smaller and less stable Taylor cone that emerges from a set feed rate, the needle and the Taylor cone may eventually merge. Fibers were thinner because the polymeric solution was stretched more by the higher voltage [26]. Researchers discovered that increasing voltage reduced the diameter of fibers formed by ES until they reached their lowest point before the trend reversed. After initially increasing with an increase in voltage, the diameter of the fibers starts to shrink [27]. The voltage causes the droplet of the polymeric solution to expand in size. Faster acceleration into a fiber collector may result from a more significant potential difference, reducing the time it takes to expand before depositing on the collector. 

The electric field intensity decides the diameter of freshly generated fibers ranging from several microns to nanometers. Through the optimization of the applied voltage, it could be achieved depending on the new solution of polymeric mixtures. Insufficient or inappropriate electric field intensity may lead to a kind of bead formation in the spun fibers or possibly to the breakdown of the jet formation. Observed that raising the voltage adjusted the geometry of the target surface at which the new formation of a Taylor cone and fiber jets are generated. However, when the applied voltage was raised, a reduction in the drop volume led to the formation of a Taylor cone at its tip; this was related to an increase in bead defects among electrospun fibers [28].

#### 2.2.2. Solution’s Flow Rate

The solution flow rate at which the newly synthesized polymeric new solution travels through the cylindrical syringe is also critical. The flow velocity of the newly synthesized polymeric solution is reduced to allow for polarization. High flow rates produce thick-diameter bead fibers rather than smooth filaments compared to Table 5. Since the tubes are very thin at low elongation of stretching, pressure and rapid drying are carried out before the polymeric solution reaches the collector. Periodic dripping and beaded fibers will occur with high input rates of newly synthesized polymeric liquid solution. Thus, several studies have detailed the formation of droplets and moist threads [29,30,31]. The electrospinning jet’s more extensive volume and starting radius decrease the bending instability and increase fiber diameter. The insufficient charge-repelling force pulls the solution away from the nozzle tip as the feed rate increases [32]. Wet fiber deposition and excess solution droplets may occur. The flow rate of the solutions decides the smoothness and thickness of the fibers.

#### 2.2.3. Role of Collectors

Collectors were used to gather charged fibers during electrospinning. Although aluminum foil is utilized as a fiber collector, transferring the nanofibers to other substrates for usage is problematic. Diverse collectors, including wire mesh, have been created to transmit fibers to parallel or gridded bars, spinning rods, or wheels.

#### 2.2.4. Role of Distance b/w Collector Tip of the Syringe

The travel distance between the syringe tip and collector influenced the fiber diameter and morphology. In summary, too short a distance prevents the fiber from solidifying before nearing the collector. If the travel distance is too long, this results in bead fibers. Dryness from the solvent is a key physical element of electrospun fibers; thus, optimal distance is advised.

### 2.3. Ambient Parameters

Aspects of the environment may impact fiber diameters and morphologies (humidity, temperature, etc.). Due to the inverse connection between solution temperature and viscosity, polyamide-6 fibers benefit from a smaller fiber diameter. The 2.15 SEM pictures of electrospun PA-6-32 fibers at temperatures A and B are both 30 °C. A and B have sizes of 98 and 90 nm. Moisture content might completely dry the solvent and speed up its evaporation rate. Increasing humidity reduces the stretching pressures and causes thicker fiber diameters. The surface morphologies of electrospun PS fibers might vary depending on humidity. 

#### 2.3.1. Polymeric Solution: Temperature

The rate of solvent evaporation rises with temperature. The second parameter is polymer solution viscosity, which decreases with temperature. The average diameter of electrospun nanofibers changes dramatically with temperature and humidity. RH can make nanofibers thicker or thinner depending on the polymer’s chemistry. The temperature change has two conflicting impacts on the average diameter. Solvent evaporation rate and solution viscosity both influence mean fiber diameter. Heat and humidity will impact other polymer systems [33]. Achieving a consistent fiber diameter is critical for a nonwoven’s performance. The influence of humidity and temperature is critical here [34].

#### 2.3.2. Humidity

The average CA nanofiber diameter rises with humidity, but the average PVP nanofiber diameter decreases. The average diameter of electrospun nanofibers changes dramatically with temperature and humidity [33]. This research found that when relative humidity rose, the diameter of the SF/PVA electrospun nanofibers reduced from 111 to 90 nm [35].

#### 2.3.3. Air Flow

Proper adjustment of factors may impact fiber morphologies. Any desired fiber morphology and diameter may be made by electrospinning. The exemplary processing conditions may produce uniform fibers with no beads, and electrospun fibers have a lower glass transition temperature than native polymer. Polymer molecular weight, solution concentration, and solvent system substantially influenced fiber diameters and morphologies [36]. The effects of various ambient parameters are shown in Table 6.

## 3. Surface Modification of the Electrospun Nanofiber

The hydrophilic functional groups present in the outer surface of the newly formed nanofibrous scaffolds were efficiently changed by plasma treatment and AA grafting. They have a significant effect on proliferation and cell adhesion when tested in vitro [37].

## 4. Electrospinning: Applications

### 4.1. Medical: Tissue Engineering 

To repair the extracellular matrix that has been damaged, a wide range of scaffold-based tissue engineering technologies and materials have been developed. During the last decade, researchers have concentrated on creating biodegradable polymeric membranes that can be used to regenerate periodontal tissue [38]. Due to their diversity in material selection and control over scaffold features, electrospun scaffolds have been employed in tendon/ligament, neural, vascular, cartilage, and bone applications. Tissue engineering (TE) is a fascinating field that combines engineering and biology to generate or repair organ tissue. It uses cells, biomaterials, and biomolecules as its primary tools. By using TE, organ transplantation may be avoided. The seeding of cells into the material structure of the artificial materials given by TE to assist 3D tissue creation is frequently enhanced. Scaffolding methods abound, but electrospinning (ES) stands out for its ability to construct nonwoven fibrous structures with dimensional components equivalent to ECM fibers. Several studies have shown that the ES approach may be a feasible scaffolding method for tissue-engineering applications [39].

A tissue substitute’s architecture has a significant role in regulating tissue development [40]. For the ideal scaffold to function, it must meet a range of frequently competing requirements, including adequate surface area, optimal levels, and sizes of porosity to enable cell migration, and degradation rates that closely mimic the regeneration rates of the desired natural tissue [41]. Even though there are many different forms of synthetic scaffolding, a few stand out as particularly promising. According to the extensive usage of the ES process, structures made of ES fibers come under this category [42]. Scaffolds created using ES imitate the natural extracellular matrix (ECM) using nano- and microfibers, making them a simple and versatile technique. Despite their many benefits and widespread usage, the limited cell infiltration and low mechanical strength of electrospun scaffolds make them unsuitable for all but the most demanding applications. A variety of research groups have addressed the restrictions as mentioned above [41].

Controlled drug release using electrospun matrices, including antibiotics, DNA, and proteins, are possible for applications in tissue engineering. To regenerate cartilage, tissue engineering has employed collagen sponges, hydrogel scaffolds, and gelatin-based microspheres. A similar structure to the ECM in native cartilage has been explored in nanofibers made from synthetic, natural, and new combinations of polymers [43]. Electrospun nanofibers may increase scaffold properties including cell-matrix interaction and chondrogenic differentiation. The large-surface-area electrospun PCL scaffolds facilitated cell-matrix interaction and chondrocyte formation without growth factors [44].

Clinically, tendon and ligament injuries, common in young athletes, provide a considerable difficulty because of their inherent inability to recover. As a result of the healing process, scar-like tissue often develops with inferior mechanical qualities. A new method for tendon and ligament regeneration is possible via tissue engineering. From the nanofibrous structure developed through the electrospinning process used for scaffolds, researchers have been able to mimic the collagen fiber bundles of natural tissues in the regeneration of ligaments and tendons [45]. The mechanical characteristics of normal healthy tendons are extremely anisotropic because they are formed of parallel arrays of tightly packed collagen fibers. Therefore, nanofiber-based scaffolds that mirror the anisotropic structure of tendon and ligament tissue are potential options for tissue engineering. The cellular behavior of stem cells and committed fibroblasts has been studied with the nanofiber’s diameter and orientation [46].

A three-dimensional multilayered scaffold developed using polyethylene-glycol-based porous chitosan and nanofibrous mats [4]. Compared to the 3D random and porous control scaffolds, the 3D-aligned nanofiber-embedded scaffold exhibited a cell filtration depth of 45 m. Consequently, cell viability on the 3D-AL scaffold was much greater than on the 3D-RD scaffold 7 days after seeding (OD value of around 2.2 and about 1.5, respectively). As opposed to random nanofiber scaffolds, tenomodulin expression was maximum when platelet-derived growth factor-BB was immobilized in a gradient, aligned nanofiber scaffold (18.501.45). In an in vivo experiment, the supraspinatus tendon reinforced with an electrospun PLGA nanofibrous scaffold exhibited a higher Young’s modulus (48.6 MPa) than the supraspinatus tendon that had just seen primary healing (3.79 MPa) [47]. We also demonstrated an electrospinning approach that used PCL scaffolds seeded with human ADSCs to improve cell penetration and collagen deposition. Compared to nonaligned multilayered scaffolds, the fold change in collagen type III and tenomodulin increased from 2 to 2.5 and 3 to 25, respectively, indicating improved tendon-related gene expression.

For a variety of cardiovascular illnesses, there is increasing need for tissue-engineered vascular grafts (TEVGs) to repair or bypass damaged arteries. To create TEVGs from tissue skeletons, biopolymers and biodegradable synthetic polymers have been used. The inability to replicate natural tissue mechanical properties and the necessity for long-term patency and development needed for in vivo function remain unclear. Scaffolds are often made by electrospinning, which has the potential to alleviate these issues. TEVGs from humans have not yet been tested using this technique. The first human trials of tissue-created blood vessels for high-pressure arteries have been completed. 

**Figure 4:**
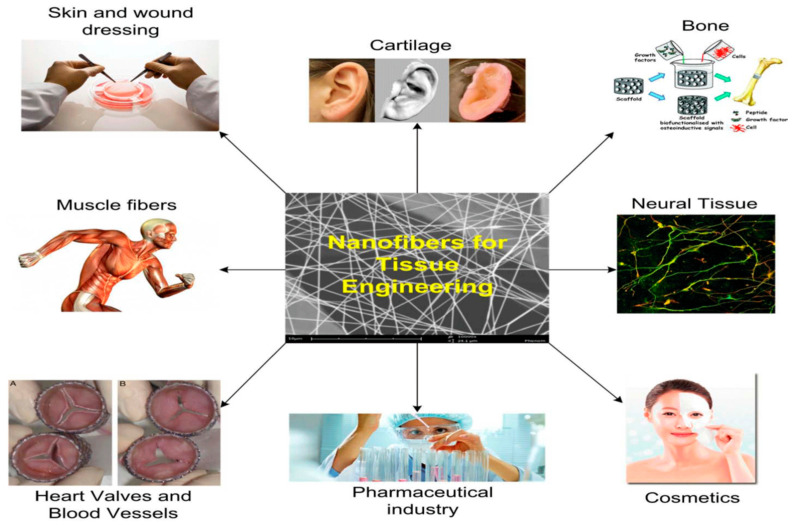
A schematic of the applications of tissue engineering [48].

Tissue-engineered scaffolds are injected into the urinary tract organs for reconstructive purposes through electrospun scaffolds in patients with a sick bladder who have lost their ability to operate as a compliant and low-pressure reservoir must undergo augmentation cystoplasty. It is possible to regenerate some lost bladder tissue using electrospun scaffolds as shown in Figure 4. An electrospun scaffold may be used to replace an affected urethral wall with a stricture lesion, either as a direct or indirect replacement.

### 4.2. Renewable Energy

Electrospinning is a simple and low-cost method for producing nanofibers. Inorganic materials, mainly metal oxides, may be created and electrospun to improve their conducting and ceramic characteristics, a massive boon for energy devices. Sustainable energy and habitats will be made possible in the future when it comes to advances in nanotechnology. Nanofiber-structured materials can be extremely effective in addressing energy, health, and environmental concerns. Innovative methods for harvesting renewable energy using cutting-edge technology are made possible by advances in nanotechnology. Semiconductors made from electrospinning nanofibers, in particular, are being touted as a potential solution to our energy woes. Electrospinning, a simple and inexpensive method for creating nanofibers, is an effective way to do so. It is also possible to create nanofibers from a wide range of materials, including organics and inorganics, using electrospinning [49].

In electrospinning, a polymer solution is charged and ejected under a high-voltage electric field in order to create micro- and ultrafine fibers (in nanometers). Polymer solution is spun into yarn using an electrospinning process governed by electrohydrodynamic principles. Electrospinning’s basic setup may be seen in the image. Here, the polymer solution is held in a reservoir (usually in the form of a syringe) and is applied to an electrode through the pump, high-voltage power source, and collection device described above [50]. Electrostatic repulsion causes the droplet to be stretched when high voltage is given to a polymer liquid droplet. A jet of liquid gushes out from under the collector at this moment. Taylor cone is the name given to the eruptive site. Taylor cone geometry is a compromise between the Maxwell stresses that are transmitted into melting fluid and the surface tension that maintains meniscal shape. In this case, the jet does not break up into droplets but creates an electrically charged laminar jet, which elongates owing to electrostatic repulsion. This is the mechanism of electro-spraying. In solution electrospinning, or in melt electrospinning, the jet dries or cools sufficiently to become solid, and a nanoscale fiber is generated (4).

Fibers with distinct structures and characteristics can be made by altering the spinneret or the solution. Using electrospinning in a number of ways is a viable option. Two solutions can be injected into the spinneret tip for co-axial electrospinning. The electrospinning jet’s Taylor cone’s sheath fluid is hypothesized to act as a carrier for the inner fluid, bringing it into contact with the outer fluid [51]. To make core-shell or composite fibers without changing the spinneret is possible in emulsions. However, because of the increased number of factors that must be taken into account while generating the emulsion, these fibers are often more difficult to make than coaxial spinning [52]. Polymer melt electrospinning, on the other hand, does not require volatile solvents because it uses polymer melts. Semicrystalline fibers are made from a variety of polymers, including PE, PET, and PP. Electrospinning with a syringe or spinneret, a high-voltage source, and a collector is quite similar to conventional electrospinning. Resistance heating, flowing fluids, air heating, or layering are the most common methods for melting polymers [53].

Electrospinning is a valuable technology for making micro- and nanoscale fibers in both laboratories and industry. Numerous polymers, including single and mixed polymers, are employed in electro-fiber production. Electrospinning’s intricate hierarchical structure under regulated calcination is one of its unique advantages [54]. There are several ways to use the unique features for energy, environment, and tissue engineering. There are a number of energy-related applications for electrospinning technology, including the production of batteries, dye-sensitive solar cells, and supercapacitors [55].

### 4.3. Face Mask

SARS-CoV-2 may be prevented from spreading from person to person by wearing a face mask made of ultrafine fibers with a diameter of a few tens of nanometers or less. For example, the electrospun ultrafine fiber filter can be antiviral, transparent, and degradable in addition to virus-blocking. This is an essential component in the battle against epidemics. Filtration performance and reusability of electrospun ultrafine fiber-based masks: a production technique [56,57,58].

**Figure 5:**
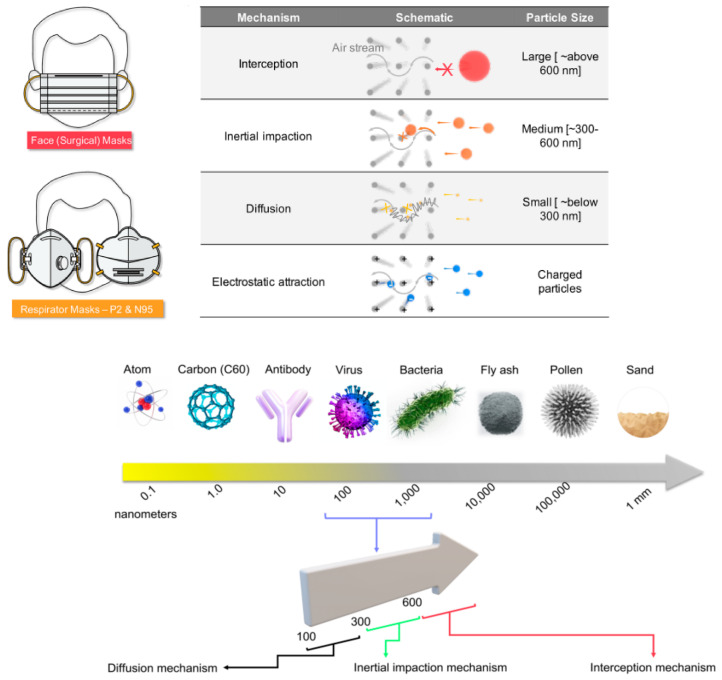
Various types of protective masks [56].

Since the dawn of humans, we have relied on textiles for everything from clothing to shelter. On the other hand, fibers are the fundamental building blocks of these substances. According to this viewpoint, nanofibers, as active layers in face masks, may protect patients against the new coronavirus sickness (COVID-19). A few things should be known about these electrospun nanofiber face masks in particular, including how they work and how you may build your own at home, all in one short, but essential, letter. Fine tiny sieves, which operate as active layers in face masks, are what give them their functionality. A microscopic sieve is composed of entangled mats comprised of extremely fine fibers capable of providing complicated routes for airborne particles, viruses, and bacteria. Three distinct particles may be prevented from reaching the user via four different techniques while the face mask is in use. Micro-, macro-, and nanoparticles are all categorized independently in Figure 5.

The three particle categories now include charged particles. Larger macroparticles (over 600 nm) cannot pass through the filters and are intercepted outside the masks. Microfine particles (300–600 nm) may pass through the mat pores of the mask sieve, but they are more likely to smash against the fiber walls (like any item traveling in a non-straight route at high speeds). The particle’s mass and velocity govern this, preventing it from reaching the wearer. This is called the impact/collision mechanism. Due to their tiny size, nanoparticles (below 300 nm) may readily pass through pores without interacting with the pore walls but are quickly assaulted by the air molecules surrounding them. Diffusion-based capture is used to capture such particles, which only occurs in finer fibers (nanofibers) and branching nanofibers.

Electrospun nanofibers are more efficient here. However, small particles (300 nm) that do not obey the impact/collision process, or the diffusion-based capture method are difficult to filter in many face masks. To delay such particles and allow them to follow one of the processes, numerous layers of mats are required. Multiple filtration layers present a new technical challenge for the final product’s breathability. Engineers must handle the opposing needs of air filterability and air breathability to achieve the perfect performance of a face mask. With proper electrospinning process management, electrospun nanofibers offer the necessary equilibrium. By using electrostatic filtration, the last process, particles attracted by their negative charges are prevented from reaching the wearer by filtration mats that are charged [57].

**Figure 6:**
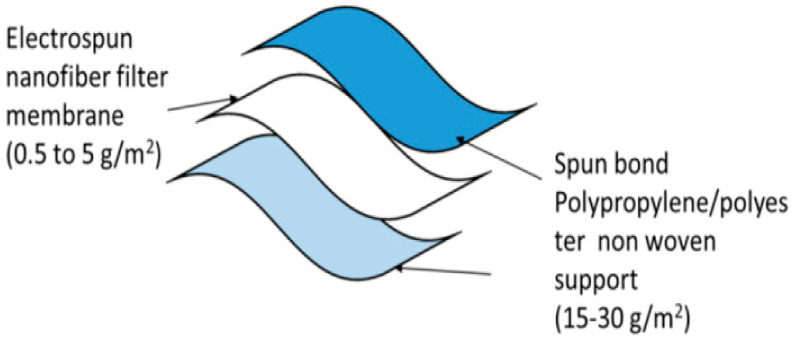
Three-layered nanofiber masks [57].

Two polyester/polypropylene nonwovens plus a fine-fiber filtration membrane make up a face mask’s three or more layers of composite structures shown in Figure 6. As an additional layer of activated carbon cloth, VOCs may be removed (Singh et al. 2002). Additionally, the wearer’s nose and mouth are protected by a layer of modacrylic to keep the mask in place and prevent it from falling apart. As a result, most commercial masks are constructed using four to five layers of cloth.

The skin contact layer should be made of soft nonwoven fabric that is hydrophilic and retains cough droplets. The outermost membrane may be a blood/liquid repellent. The middle layer membrane is a high-efficiency performance filter membrane with tiny pores [58]. The requirements of the suggested masks are listed in Table 7.

**Figure 7:**
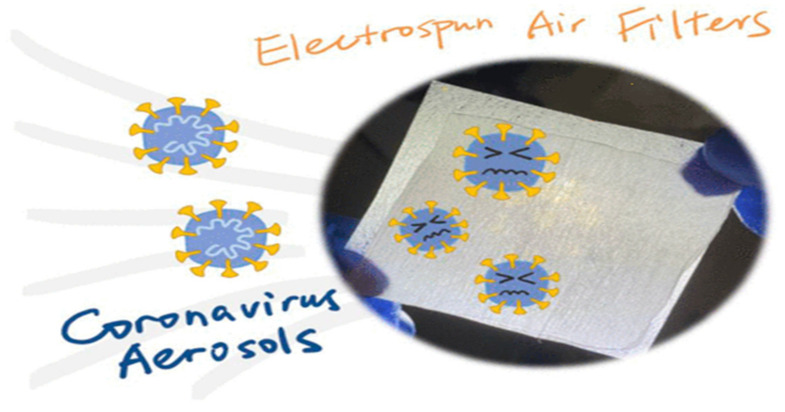
Coronavirus aerosols [59].

SARS-CoV-2 airborne transmission is essential in COVID-19 dissemination. We created electrospun nanofibrous air filters for personal protection equipment and the interior environment to safeguard public health. The electrospun air filters featured substantially smaller pores than surgical and textile masks due to ultrafine nanofibers (300 nm) (a couple of microns versus tens to hundreds of microns). In prior investigations, coronavirus strains were utilized to create aerosols for filtering efficiency testing as shown in Figure 7. The electrospun air filters surpassed several conventional face masks by catching up to 99.9% of coronavirus aerosols. A comparison of the filtration effectiveness of coronavirus aerosols and NaCl aerosols was also performed. It was shown that NaCl aerosols might be employed as an effective surrogate for coronavirus aerosols in filtering experiments using air filters and face masks of various sizes and efficiency. To stop SARS-CoV-2 transmission, we developed electrospun nanofibrous air filters. It is also possible to learn how air filters catch coronavirus aerosols by studying the removal effectiveness of NaCl [59].

### 4.4. Melt Electrospinning

**Figure 8:**
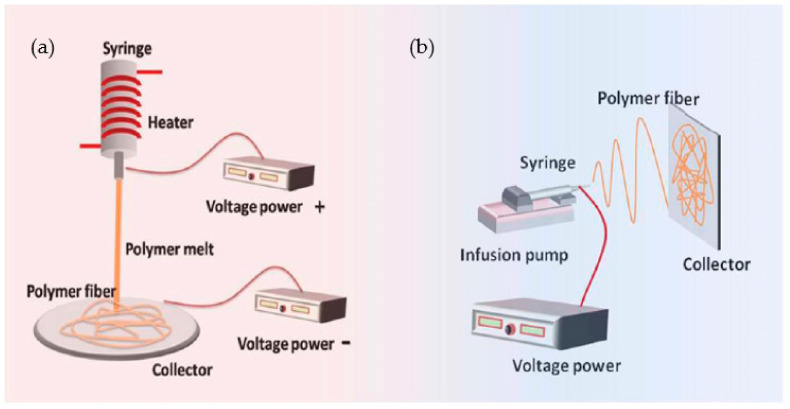
(**a**) Melt and (**b**) Solution Electrospinning [62].

Melt electrowriting is a new form of an additive manufacturing (AM) process which is used to fabricate new thermoplastic microfiber scaffolds used in tissue engineering [60]. It is presently a newly emerging technique used for fabricating micro-/nanofibers. It could precisely control the deposition of newly synthesized polymer microfibers through electrohydrodynamic jet deposition on the surface collector via a computerized controlled collector [61]. Melt electrospinning produces different fibrous shapes and structures from new combinations of polymeric solutions for different applications such as textiles, filtration, and tissue engineering. The PCL melt electrospinning (ME) is shown in the fibers in Figure 8a produces a smooth and high crystallinity, whereas the PCL solution electrospinning (SE) is shown in Figure 8b produce fibers exhibiting a high porous and more amorphous nature than the PCL ME fibers. The effectiveness of different process parameters of both the ME and SE techniques used for developing morphology and fiber diameters were researched [62]. Table 8 presents the comparison of drug release using melt electrospinning and solution electrospinning.

The PCL melt fibers were a more suitable solution in fibers (Lian and Meng 2017). The morphological analysis revealed high porosity of solution/melt electrospun mats (94.78%). Higher filtration efficiencies were reached when both electrospinning techniques were applied simultaneously [64]. The melt electrospinning writing (MEW) was used to fabricate scaffolds with good mechanical strength and controllable structure and for regeneration of bone. The gelatin nanofibers were incorporated in scaffolds to become hydrophilic and enhance the mechanical strength [59].

MES is a new method for developing amorphous and water-soluble drugs. If the water contents were better controlled, then it would enable minimizing the MES operating temperature, significantly enhance the plasticization and develop extra pressure in a cylindrical syringe, which minimizes thermal depletion of the incorporated drug [65]. The ME eliminated the use of aggressive solvents and sulfonation which is toxic to PEEKs. This process could replace the current solution fabrication techniques used in the various fields for PEEK fiber membranes [66].

### 4.5. Melt Electrowriting

A random copolymer setup with controlled molecular weights and different compositions enables fabricating the well-defined structures [67]. Melt electrowriting (MEW) is an additive fiber-manufacturing process that is capable of fabricating thin-microfiber thermoplastic-based scaffolds for tissue engineering. It produces micron-scale biocompatible constructs through electrodynamic jet deposition with a high level of orientation control over fiber deposition on the collector. The rotating cylindrical collector to produce the tubular geometry could fabricate the anatomical tissues such as blood vessels [68].

### 4.6. Wound Dressing

Electrospun wound dressings are among the most prominent. Wound-healing medications can be incorporated into these dressings in a variety of ways, depending on the patient’s specific needs [69]. Electrospinning can be used to create a wound dressing that mimics the skin’s natural extracellular matrix (ECM) in order to enable cellular communication, adhesion, migration, and proliferation, as well as cellular adhesion, migration, and proliferation. It is possible to produce submicron fibers with a high surface-to-volume ratio that resembles the structural features of ECM using electrospinning as an alternative method [70].

Electrospun nanofiber mats are capable of filtering even the tiniest particles or molecules, thanks to their extremely narrow pores. Because of this, they can be used in conjunction with other materials, such as those with antibacterial properties or high surface-to-volume ratios, to further treat the filtered material. Fine nanofiber mats are susceptible to mechanical damage; however, reinforcement options include inserting them in composites or attaching them to more mechanically stable layers. Stabilizing electrospun nanofiber mats by 3D printing hard polymer layers on top of them is generally feasible. In order to avoid delamination of the nanofiber mat and damage to it by the hot nozzle, here we report on the reversed process (i.e., first 3D printing a rigid scaffold and then electrospinning the nanofiber mat on top of it), which is more difficult to perform [71]. The antibacterial activities increased with increasing ZnO nanoflower contents, and these 3D-printing filaments were better against Gram-positive than Gram-negative bacteria because of differences in their cell walls [72,73].

## 5. Conclusions and Future Directions

The different process parameters were studied and found their significant roles during the electrospinning process. The solution parameters play important roles such as optimum concentration (uniform nanofibers), higher molecular weight (smoother fibers), higher conductivity (smaller and uniform nanofiber diameter), and optimal viscosity (constant ejection from jet/needle). Additionally, process parameters control polymer ejection, fiber smoothness, and better connectivity as follows: higher applied voltage (14–30 kV), slow feed rate, and optimum tip-to-collector distance (13–15 cm). Ambient parameters such as operating temperature and humidity also play significant roles in controlling the thickness/thinness of the nanofibers. The nanofibers produced through electrospinning process could be used in wider areas including tissue engineering, biosensors, medical devices, wound dressing, cable for implantable devices, fuel cells, drug-coated stents, neural prostheses, artificial heart valves, air filters, water filters, engine filters, gas turbine filters, personal protective masks etc.

## Figures and Tables

**Table 1:** Solutions and characterization methods of different polymers developed by electrospinning.

Polymers	Solvents	Characterization Methods
PEO/Lignin	Methanol and DCM	FTIR, SEM, SEM, XRD, DSC, mechanical test, TGA, in vitro and in vivo
CS/PEO	Acetic acid/DI water	
PEO	DI water	
PVA	DI water	
PAN/PMMA	DMF	
PMMA	DMF	
PCL	Acetone DCM/methanol HFP	
Gelatin/PCl	TFE	
PVP, PLA	Ethanol DCM and DMF	
PLGA	Chloroform/DMF	
Nylon-6	FA	
PDLLA	Chloroform	
PVDF	DMAc DMSO/acetone	
PVDF-HFP	DMF/acetone	
PPY	DMF	
PS	DCM/ethanol DMF/THF	
PLI	DMF	
PAA	DMAc/DMSO DMEA	
Collagen	HFP	
CA	HFP acetone/DMAc	

**Table 2:** Electrospinning Process Parameters [19].

Solution Parameters	Process Parameters	Ambient Parameters
**Concentration**: Optimum Concentration for uniform nano fibers **Molecular Weight**: High molecular weight for smoother nanofibers. **Conductivity**: Higher conductivity for smaller and uniform fiber diameter. **Viscosity**: Optimal viscosity for constant ejection from jet needle.	**Applied Voltage**: Higher voltage provided more polymer ejection. **Feed Rate**: Slow flow rate enhance smooth fiber production. **Tip-to-collector distance**: Optimum distance for better interconnectivity.	**Humidity**: Higher humidity results in thicker fiber production. **Temperature**: Increasing temperature generates thinner nanofibers.

**Table 3:** Comparison of the different concentrations [23].

Sl. No	Concentration	Formation of Fibrous Structure
1	Concentration level is very low	(i) Micro- or (ii) nanopolymeric fibers
2	Concentration is a little higher	Combinations of beads and fibers would be formed on macro- and nanofibers
3	Optimum and most suitable	Smooth macro- and nanofibers
4	Concentration is very high	Non-nanoscaled and helix-shaped microribbons

**Table 4:** SEM microstructure of the different fiber structures [23].

Sl. No	Molecular Weight (g/mol)	Fibrous Structure	Diameter	Cross section of the Fibers
1.	9000–13,000	Unstable and with a bead-on-string structure	250 nm and 1 Am	Circular
2.	13,000–23,000	Fibrous structure is stabilized	500 nm and 1.25 Am	Circular
3.	31,000–50,000	Flat fibers	1–2 Am	The progressive transition from circular to flat strands
4	Too high a molecular weight with the low concentration	Microribbon	-	Microribbon
5	Very high molecular weight	Helical fibers	-	-
6	Low concentration	Zigzag ribbon	-	-

**Table 5:** Observations of smooth and bead fibers concerning the effect of flow rate.

Sl. No	Flow Rate	Fibers’ Shapes	Operating Parameter
1.	Lower flow rate	Smooth fiber	Flow rates of 0.40 mL/h
2.	Very high flow rate	Thick diameter of bead fibers	Flow rates 0.66 mL/h

**Table 6:** Solution parameter and its effects.

Sl. No	Solution Parameters	Effects on Fiber	Mechanism
1	Conductivity of the Solution	It has been demonstrated that increasing the solution conductivity improves the fiber quality, as seen by fewer beads and a smaller fiber diameter.	Increased solution conductivity results in increased stretching of the solution jet due to the solution’s larger charge-carrying capacity. Additionally, increasing solution conductivity results in an increase in bending instability and a longer jet path.
	Solution viscosity	Fiber diameter will increase as the viscosity of the fluid increases.	There is a possibility that the charges used to start spinning will not be enough to stretch the solution to reach the target when viscosity increases.
2	Solvent volatility	The location with the greatest solvent volatility must be identified. Ribbon/flat fibers and fibers with surface pores may be produced by using a more volatile solvent.	Wet fibers, fused fibers, or no fiber collection can all occur when a solution is made using a solvent with a low volatility. The solidification of the new polymeric solution at the spinneret tip might cause intermittent spinning when the polymer has a high volatility.
3	Humidity	Larger fiber diameters have been linked to both higher and lower relative humidity levels.	There are two factors that contribute to this: high humidity causes polymer precipitation, preventing fiber elongation; and lower relative humidity causes faster solvent vaporization, which causes an increase in solidification rate that results in larger fiber diameter, thus resulting in thicker fibers.
4	Temperature	The diameter of the fiber will be smaller at higher temperatures.	Higher temperatures result in quicker solvent evaporation and a lower viscosity of the polymer solution.

**Table 7:** Requirements of the suggested mask [58].

Sl. No	Present Scenario	Different Suggested Mask	Mask Requirements
1	Pedestrians	Cloth/FFP 1,2 with carbon fabric (reusable)/dust mask	Respiratory protection; decrease in particulate matter and volatile organic compounds (VOCs)
2	Workers in industry	P95/N95 respirator mask with carbon fabric	Respiratory protection is required to reduce aerosols, emissions/VOCs, gaseous
3	Pandemic	3-ply fabric mask	speaking or breathing without a valve, reduce the ejection or aerosol, inhalation or cough droplets
4	Infected persons	Medical/N95 mask	PM, cough droplets, biological aerosol, no valve, respiratory protection, and source management are all addressed in this treatment
5	Healthcare	Medical mask	Cough droplets and blood are filtered out by biological aerosol
6	Doctors	Medical N95/surgical respirator mask	Filter cough droplets

**Table 8:** Comparison of of Drug Release Using Melt Electrospinning and Solution Electrospinning [63].

Sl. No	Melt Electrospinning	Solution Electrospinning	References
1	High crystalline and without a burst phase.	Low crystallinity, porous structure, rough, high drug release rate with a burst	[63]
2	Diameter of fiber is 1.92 ± 3.31 m when TTCD is 60 mm	Diameter fiber of 0.37 ± 0.34 m when TTCD is 200 mm	[62]
3	With lower filtration efficiencies	Higher filtration efficiencies	[64]
4	Microfibrous with grid structure	Morphology of random nanofiber	[59]

## Data Availability

Not applicable.
